# Supplementary material for: The Anti-Inflammatory Response of Lavandula luisieri and Lavandula pedunculata Essential Oils
Source: Plants (Basel). 2022 Jan 29;11(3):370. doi: 10.3390/plants11030370 (PMC8838270; doi:10.3390/plants11030370)
Supplement: Supplementary file 1 [file plants-11-00370-s001.zip › Figure S1 with caption.pdf]

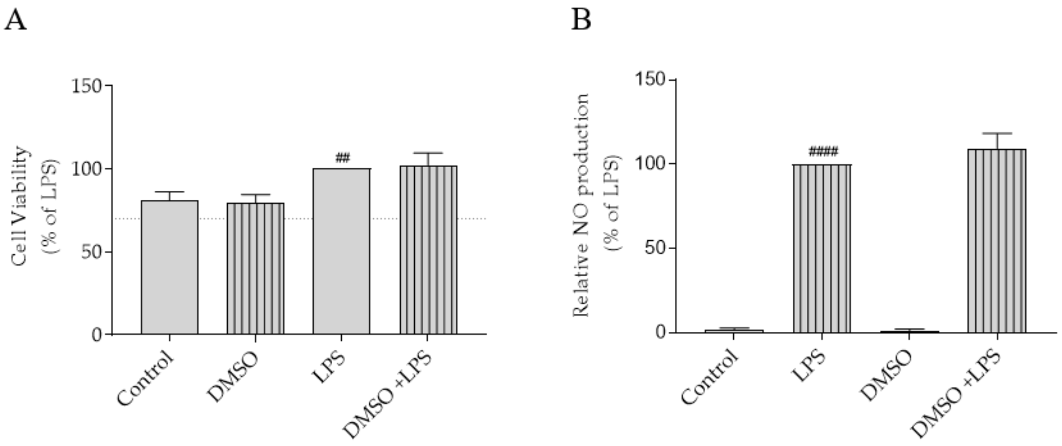

Figure S1. Effect of DMSO on A) macrophages viability and B) NO production. Cells were maintained in culture medium (control) alone or with DMSO (0.1%), or stimulated with 1 μg/mL LPS alone or in combination with DMSO (0.1%), for 24 h. Cell viability results are expressed as percentage of MTT reduction by cells treated with LPS. Nitrite concentration was determined from a sodium nitrite standard curve and the results are expressed as a percentage of NO production by cells treated with LPS. Each value represents the mean ± SEM of three experiments, performed in duplicate (##p<0.01 and ####p<0.0001, compared to Control). The dotted line in cell viability graphs represents the threshold (70% of maximal viability) below which cytotoxicity is recognized, in agreement with standard ISO 10993-5.
